# Physiological Effects of a Tallow-Incorporated Diet Supplemented With an Emulsifier and Microbial Lipases on Broiler Chickens

**DOI:** 10.3389/fvets.2020.583998

**Published:** 2020-09-22

**Authors:** Samiru Sudharaka Wickramasuriya, Shemil Priyan Macelline, Hyun Min Cho, Jun Seon Hong, Seung Hwan Park, Jung Min Heo

**Affiliations:** ^1^Department of Animal Science and Biotechnology, Chungnam National University, Daejeon, South Korea; ^2^Faculty of Science, School of Life and Environmental Sciences, The University of Sydney, Sydney, NSW, Australia; ^3^SNH Biotech Co., Ltd., Daejeon, South Korea

**Keywords:** blood metabolites, broiler, emulsifier, growth performance, gut health, lipase, tallow

## Abstract

The aim of this study was to investigate the effect of dietary emulsifiers and lipase supplementation on growth performance, blood metabolites, intestinal organ weight, gut morphology, nutrient digestibility, carcass measurements, and meat quality in broiler chickens. A total of 384, 1-day-old Ross 308 broiler chicks were randomly allocated to one of eight dietary treatments arranged in a completely randomized design with 6 replications per treatment and 8 birds per cage. Diets were corn-soybean meal-based and formulated to meet the nutritional requirements for Ross 308 specifications. Beef tallow used as the fat source in all diets. Dietary treatments were as follows, (1) positive control (PC; energy sufficient diet); (2) negative control (NC; energy deficient,−100 ME, kcal/kg); (3) NC+POL (0.1%, Polysorbate-20); (4) NC+CET (0.1%, Ceteth-20); (5) NC+POL+TLL (0.1%, *Thermomyces lanuginosus* lipase); (6) NC+POL+CRL (0.1%, *Candida rugosa* lipases); (7) NC+CET+CRL and (8) NC+LL (0.05%, Lysolecithin). Growth performances were measured weekly. One bird per pen was selected and sacrificed to collect blood, ileal digesta, jejunum sample, viscera organ weight, and meat samples on day 21 and 35. Results revealed that birds fed NC+POL+CRL diet had higher (*P* < 0.05) body weight, weight gain, and the improved (*P* < 0.05) feed efficiency compared to birds fed other low energy diets, and the effect was more prominent at the grower phase from day 21 to 35. Similarly, higher (*P* < 0.05) villi height and lower (*P* < 0.05) crypt depth commensurate with higher (*P* < 0.05) V:C ratio were observed with the broiler chickens fed NC+POL+CRL diet compared to broiler chickens fed NC diet on day 21 and 35. Moreover, broiler chickens fed NC+POL+CRL diet showed improved fat and energy digestibility compared NC diet counterpart on day 35. This study, therefore indicated that Polysorbate-20 together with *Candida rugosa* lipases had promising ability to improve growth performance of broiler chickens fed with low energy diet and curtail the growth depression without affecting blood metabolites, carcass, and visceral organs weights.

## Introduction

With the increasing price of feed ingredients and the associated energy cost, feed formulations are utilizing alternative energy sources (i.e., cheaper sources). As an alternative low-cost option, dietary fat and oil can provide a reasonable level of energy in the diet. However, the quantity of fat and oil added to a diet should be restricted to no more than 5% because a higher fat and oil content could negatively impact both the feed manufacturing process and feed quality (1). According to previous studies (2, 3), different types of fat influence the growth performance of fast-growing broiler chickens. Nevertheless, impeded fat digestion and absorption from the feed matrix occurs because of the incompletely developed digestive tract of young broiler chickens (4). As a remedial measure, the practice of supplementation with exogenous emulsifiers has become commonplace in the feed industry. Synthetic and natural emulsifiers such as milk-derived casein, calcium stearoyl-2-lactylate, and sodium stearoyl-2-lactylate have been tested in broiler diets (5–7).

To date, limited studies have been well-conducted with exogenous emulsifiers, and inconsistent responses in broiler chickens have been noted. Only a few studies have reported improved growth performance (i.e., body weight, daily gain, and feed efficiency), and nutrient digestibility of broiler chickens (8, 9). Contrarily, it has been reported (6, 10) that emulsifiers have no significant impact on the growth performance of broiler chickens. The variation in the efficacy of exogenous emulsifiers could be attributed to many factors such as fat type, bird age, lipase activity, and hydrophilic-lipophilic balance.

Supplementation with exogenous lipases either alone or in combination with emulsifiers is another approach that can be used to improve lipid digestibility in broiler chickens. Lipases are known as triacylglycerol acylhydrolase enzymes that function as catalysts in long-chain acylglycerol hydrolysis at the water-lipid interface (11). With the advancement in biotechnology and process development, microbial lipase production has become more cost-effective. This gradual price reduction of exogenous lipases is of interest for its practical application in broiler diets. According to previous studies, broiler chickens fed a diet supplemented with exogenous lipases showed an improvement in growth performance and nutrient digestibility (12, 13). In contrast to these observations, a study conducted previously (14) demonstrated no significant improvement in broiler performance as a result of lipase supplementation. Similar to the studies on emulsifiers in broiler diets, the above-mentioned results are inconsistent and inconclusive.

Polysorbate-20 and Ceteth-20 are surfactants and are widely used as emulsifying agents, solubilizing agents for cosmetic preparations, cleansing agents, flavor dispersants, and dough improvers in a range of industrial applications, such as pharmaceutical preparations and fruit or vegetable coatings (15). *Thermomyces lanuginosus* lipase (TLL) and *Candida rugosa* lipase (CRL) are two commercially available enzymes produced as a consequence of advances in biotechnology. A plethora of examples of the properties of these emulsifiers and lipases and their applications have been fully documented. However, no one reported about the prospective dietary applications of these emulsifiers (POL and CET) and lipases (CRL and TLL) in the animal feed industry.

Therefore, the present study was conducted to investigate the effects of the emulsifiers Polysorbate-20 and Ceteth-20; and the lipases TLL and CRL on growth performance, blood metabolites, visceral organ weights, small intestine morphology, nutrient digestibility, carcass measurements, and meat quality in broiler chickens fed corn-and-soybean-meal-based diets containing tallow as it wasn't reported previously. The hypothesis tested in this study was that gut health and nutrient digestibility would improve in broilers fed a low-energy diet supplemented with emulsifiers and microbial lipases, thus maintaining growth performance and improving meat quality.

## Materials and Methods

### Birds and Housing

An experiment was conducted using 384 Ross broilers from day 1 to 35 days of age. Eight birds were housed in each raised wire-floor pens (0.85 × 0.55 × 0.35 m^3^), with similar mean body weight (40.24 ± 0.25 g; mean ± SEM) and weight distribution. Each pen was equipped with two nipple drinkers and a metal trough. Birds were offered the experimental diets on an ad-libitum basis for 35 days. Birds had free access to fresh clean drinking water *via* nipple drinkers throughout the experiment. All the management practices were followed by Ross 308 broiler management guidance (16).

### Experimental Design and Diets

Birds were allocated to one of eight dietary treatments (Table 1) arranged in a completely randomized design with 6 replicate pens per each treatment. Two basal diets (PC, Energy sufficient positive control vs. NC, 100 kcal/kg lower energy negative control) were formulated based on corn and soybean meal (Table 2), to meet the nutrient requirements of Ross broiler 308 nutrient specifications (17). Beef tallow was used as a fat source in the experimental diets. The basal diets were not contained any antimicrobial growth promoters or alternatives. Two-phase feeding was followed as starter and grower phases. The negative control diet was used to produce the other six emulsifier treatment diets. The basal diet was supplemented with emulsifier alone or together with lipase accordingly to produce other treatment diets as shown in Tables 2, 6. Meantime, Cr_2_O_3_ (Daejung chemicals and metals Co., Ltd. Gyeonggi-do, Korea) was added as an index for digestibility analysis in a proportion of 0.3% to all eight experimental diets.

**Table 1:** Eight different dietary treatments of the experimental design^1^.

**Treatment**	**Description**
PC	Positive control diet with standard nutrient levels (Ross 308, nutrient specifications)
NC	Negative control with 100 kcal reduce energy diet
NC+POL	NC supplemented + 0.1% Polysorbate-20
NC+CET	NC supplemented + 0.1% Ceteth-20
NC+POL+TLL	NC supplemented + 0.1% Polysorbate-20 + 0.1% TLL^1^
NC+POL+CRL	NC supplemented + 0.1% Polysorbate-20 + 0.1% CRL^2^
NC+CET+CRL	NC supplemented + 0.1% Ceteth-20 + 0.1% CRL
NC+LL	NC supplemented + 0.05% Lysolecithin

1 *Thermomyces lanuginosus lipase (SnH Biotech Co., Ltd. Yuseong-gu, Daejeon, Republic of Korea)*.

2 *Candida rugosa lipase (SnH Biotech Co., Ltd. Yuseong-gu, Daejeon, Republic of Korea)*.

**Table 2:** Composition (g/kg, as-fed basis) of the experimental diets.

**Ingredients**	**Positive control**		**Negative control**	
	**1–21 days**	**22–35 days**	**1–21 days**	**22–35 days**
Corn	50.00	60.60	45.28	56.47
Wheat bran	3.67	-	9.29	5.37
Soybean meal, 48%	37.50	30.81	36.60	29.56
Beef tallow	4.50	4.50	4.50	4.50
Limestone	1.50	1.50	1.50	1.50
Mono-calcium phosphate	1.70	1.50	1.70	1.50
Iodized salt	0.35	0.30	0.35	0.30
Vit-Min premix^1^	0.30	0.30	0.30	0.30
Lysine-HCl	0.20	0.20	0.20	0.21
DL-methionine	0.28	0.29	0.28	0.29
**Calculated values^2^**				
ME, kcal/kg	3051	3200	2950	3101
Crude protein, %	23.1	20.2	23.1	20.1
Crude fat, %	6.65	6.81	6.67	6.84
Calcium, %	1.09	1.02	1.09	1.02
Available phosphorus, %	0.49	0.43	0.50	0.44
Total lysine, %	1.38	1.19	1.38	1.19
Total methionine, %	0.63	0.59	0.64	0.61
Total cysteine, %	0.38	0.34	0.39	0.34
Total met+cys, %	1.01	0.94	1.03	0.95
Total threonine, %	0.91	0.78	0.87	0.76
Total tryptophan, %	0.27	0.23	0.28	0.23
Total valine, %	1.07	0.93	1.07	0.92
Total arginine, %	1.58	1.35	1.59	1.34
**Analyzed values**				
GE, kcal/kg	4235	4244	4203	4123
Crude protein, %,	22.78	18.81	22.89	18.62

1 *Supplied per kilogram of total diets, Fe (FeSO_4_·H_2_O), 80 mg; Zn (ZnSO_4_·H_2_O), 80 mg; Mn (MnSO_4_·H_2_O) 80 mg; Co (CoSO_4_·H_2_O) 0.5 mg; Cu (CuSO_4_·H_2_O) 10 mg; Se (Na_2_SeO_3_) 0.2 mg; I, (Ca(IO_3_)·2H_2_O) 0.9 mg; vitamin A, 24,000 IU; vitamin D_3_, 6,000 IU; vitamin E, 30 IU; vitamin K, 4 mg; Thiamin, 4 mg; Riboflavin, 12 mg; Pyridoxine, 4 mg; Folacine, 2mg; Biotin, 0.03 mg; Vitamin B_8_, 0.06 mg; Niacin, 90 mg; Pantothenic acid, 30 mg*.

2 *The values are calculated according to the values of feedstuffs in NRC (1994)*.

Microbial lipases (TLL and CRL) used in this study was pre-determined based on the *in-vitro* study (Data not shown). The added TLL has contained 3,000 lipase units per gram as enzyme activity and CRL 1,000 lipase units per gram as enzyme activity. One lipase unit was defined as the amount of enzyme activity need to release 1 μmol of fatty acid per minute under the assay condition (13). All diets mixed properly and diet samples were obtained separately from the mixer for composition analysis.

### Growth Performance

Pen basis initial body weights of the birds were recorded, which divided similarly to maintain replicate and treatment uniformity of the experiment on day 1. Thereafter, body weight and feed intake were measured on day 7, 14, 21, 28, and 35. Based on the measured body weight and feed intake data, average daily gain, average daily feed intake (mortality corrected), and feed conversion ratio were calculated on a pen basis.

### Sample Collection

Sample collections were carried at day 21 and day 35 of the experiment. One bird (closer to the mean body weight) was selected from each cage at a time (6 birds for each treatment) for sample collection. Live body weight of the bird was measured, euthanized *via* cervical dislocation, and sacrificed by cutting the carotid artery and jugular vein. Blood, intestinal organs, gut samples, ileal contents, and carcass portions were collected in each sample collection.

Before euthanizing the selected birds, blood samples were collected into two vacutainer tubes (4 mL for each) from the Jugular Vein. Vacutainers were contained spray-coated silica and a polymer gel for serum separation (BD Vacutainer® SST™, Franklin Lakes, NJ, USA). Collected blood samples were quickly transferred to a laboratory for serum separation.

After sacrificed the bird, abdominal incisions were made and separated the ileum and jejunum from the gastrointestinal tract. The ileum defined as the segment of the small intestine which extended from Meckel's diverticulum to the ileocecal junction and the jejunum was defined as the segment in-between Meckel's diverticulum to Duodenum. A 3 cm piece of jejunum was removed from the Meckel's diverticulum end and flushed with ice-cold phosphate-buffered saline (PBS saline) at pH 7.4. The sample was placed into plastic containers contained 10% formaldehyde for fixation and stored until mucosal morphology measurement analysis. Following the separation of jejunum samples, the digesta of the ileal segment was gently removed by finger stripping into labeled plastic containers. Samples were quickly stored in a freezer at −20°C until further analysis. Afterward, visceral organs (gizzard, pancreas, liver, spleen, and bursa of fabricius) were removed separately and weighed. The contents of the gizzard were removed manually and recorded the weights.

The abdominal fat pad was excised gently from the sacrificed bird and measure the abdominal fat weight. Skinless Breast muscle (including pectoralis major and minor) and whole leg (right) were removed and weighed. Thereafter, breast muscle samples were collected for meat quality analysis.

### Sample Preparation and Laboratory Analyses

Collected blood samples were centrifuged (Micro 12, Hanil Science Co., Ltd., Korea) at 3,000 × g for 10 min at 4°C. Serum samples were separated and stored at −20°C until analysis. Serum cholesterol, lipase, triglyceride, low-density lipoprotein, high-density lipoprotein, and creatinine level were analyzed using Biochemistry Analyzer 7020 (HITACHI, Tokyo, Japan).

Jejunum samples which fixed in the 10% formaldehyde were undergone the sample preparation process as described elsewhere (18). Ring-shaped lengths of ileum were excised, dehydrated, and embedded in paraffin wax. From each of these, 6 transverse sections (4–6 μm) were cut, stained with hematoxylin and eosin, and mounted on glass slides. The height of 10 well-oriented villi and their associated crypts were measured using NIS-Elements Viewer software (Version, 4.20; NIS Elements, Nikon, USA) with an inverted microscope (Eclipse TE2000, Nikon Instruments Inc., Melville, NY 11747-3064, USA) using a calibrated eyepiece graticule.

The digesta samples were pre-dried at 55°C for 24 h, ground through a 0.75-mm sieve (ZM 200 Ultra-Centrifugal Mill, Retsch GmbH & Co., KG, Haan, Germany), and analyzed for levels of dry matter, crude protein (N × 6.25, macro-Kjeldahl), ether extract, and gross energy according to the standard methodologies (19). Chromium oxide concentration of the samples was also analyzed (20). The digestibility of nutrients was calculated as described (7) using the following equation.

Apparent digestibility of a nutrient = 1 − [(M_diet_ × N_digest_) / (M_digest_ × N_diet_)]

M_diet_ is the concentration of an indigestible marker in the diet; N_digest_ is the nutrient concentration in ileal digesta; M_digest_ is the indigestible-marker concentration in ileal digesta; and N_diet_ is the nutrient concentration in the diet.

### Statistical Analyses

Data were analyzed as a completely randomized design, using the general linear model (GLM) procedure of one-way ANOVA of SPSS software (Version 24; IBM SPSS 2016). A pen used as the experimental unit for all growth performance measurements. Selected individual birds were considered as experimental units for blood parameters, visceral organ weights, gut morphology, and nutrient digestibility parameters. Mean differences observed in the treatment were considered significant at *P* < 0.05. When treatment effects were significant (*P* < 0.05), means were separated using Tukey multiple range test of SPSS software (Version 24; IBM SPSS, 2016). Pair-wise comparisons between means were made using Fisher's protected LSD test analysis procedure of SPSS software (Version 24; IBM SPSS, 2016).

## Results

### Growth Performance

The effect of emulsifier and lipase supplementation on the body weight of the broiler chickens from hatch to 35 days of age is presented in Table 3. During the first 21 days, we did not observe any body weight difference (*P* > 0.05) among dietary treatments. However, broiler chickens fed with NC+CET+CRL diet was shown lower (*P* < 0.05) body weight compared to broiler chickens fed PC and NC+POL+CRL diet on day 28 and 35. At the end, broiler chickens fed NC+POL+CRL diet was observed for the highest (*P* < 0.05) body weight compared to those fed the other treatment groups.

**Table 3:** Effect of emulsifier and lipase supplementation in diet on body weight (g) of broiler chickens^1^^,^^2^^,^^3^.

**Treatment**		**Day 7**	**Day 14**	**Day 21**	**Day 28**	**Day 35**
PC		156.38	458.73	948.44	1493.34^b^	2195.61^b^^c^
NC		160.27	452.36	912.83	1465.20^a^^a^	2048.87^a^^b^^c^
NC+POL		153.86	447.90	935.10	1430.20^a^^b^	2113.42^a^^b^^c^
NC+CET		157.50	447.38	939.67	1387.26^a^^b^	2039.71^b^^c^
NC+POL+TLL		158.98	446.11	913.81	1389.16^a^^b^	2058.95^a^^b^^c^
NC+POL+CRL		160.36	462.25	949.71	1483.87^b^	2216.41^c^
NC+CET+CRL		158.69	452.03	925.44	1351.43^a^	2011.86^a^
NC+LL		153.56	443.23	929.58	1437.45^a^^b^	2068.02^a^^b^^c^
SEM^4^		1.093	3.397	6.492	11.061	16.054
P value		0.666	0.885	0.810	0.004	0.002
Contrast	NC vs. PC	0.389	0.656	0.194	0.457	0.009
	NC vs. NC+POL	0.159	0.755	0.413	0.356	0.236
	NC vs. NC+CET	0.539	0.728	0.373	0.044	0.865
	NC vs. NC+POL+TLL	0.775	0.662	0.974	0.049	0.852
	NC vs. NC+POL+CRL	0.985	0.490	0.179	0.621	0.003
	NC vs. NC+CET+CRL	0.725	0.981	0.642	0.004	0.494
	NC vs. NC+LL	0.142	0.524	0.537	0.464	0.723

1 *Values are the mean of 6 replicates per treatment*.

2 *PC, Positive control diet with standard nutrient levels; NC, Negative control with 100 kcal reduce energy diet; POL, Polysorbate-20; CET, Ceteth-20; TLL, Thermomyces lanuginosus lipase; CRL, Candida rugosa lipase*.

3 a-c *Means within a same column with no common superscript differ significantly (P < 0.05)*.

4 *Standard error of mean*.

Similar to the body weight data, the average daily gain of the broiler chickens did not show any difference (*P* > 0.05) during the starter period up to 21 days of age (see Table 4). On day 28 and 35, birds fed NC+POL+CRL diet was shown higher (*P* < 0.05) daily gain and it was similar (*P* > 0.05) to birds fed PC diet. Considering the grower period (21–35 days) and overall period (0–35 days), birds fed NC+POL+CRL diet showed the higher (*P* < 0.05) daily gain among treatment groups while the lower (*P* < 0.05) was observed with bird fed NC+CET+CRL diet.

**Table 4:** Effect of emulsifier and lipase supplementation in diet on body weight gain (g/d) of broiler chickens^1^^,^^2^^,^^3^.

**Treatment**		**Day 7**	**Day 14**	**Day 21**	**Day 28**	**Day 35**	**Day 1–21**	**Day 22–35**	**Day 1–35**
PC		17.16	43.19	69.96	77.84^b^	100.32^b^	43.25	89.09^b^^c^	61.58^b^^c^
NC		16.60	41.73	66.81	75.67^a^^b^	83.38^a^	41.55	79.52^a^^b^	57.39^a^^b^^c^
NC+POL		16.25	42.01	69.60	70.73^a^^b^	97.60^a^^b^	42.62	84.17^a^^b^^c^	59.24^a^^b^^c^
NC+CET		16.75	41.41	68.60	68.30^a^^b^	93.21^a^^b^	42.83	80.76^a^^b^^c^	57.13^a^^b^
NC+POL+TLL		16.98	41.02	66.00	71.32^a^^b^	95.68^a^^b^	41.60	83.50^a^^b^^c^	57.68^a^^b^^c^
NC+POL+CRL		17.14	43.13	69.64	76.31^b^	104.65^b^	43.30	90.48^c^	62.17^c^
NC+CET+CRL		16.92	41.91	67.63	60.86^a^	94.35^a^^b^	42.15	77.60^a^	56.33^a^
NC+LL		16.18	41.38	69.48	72.55^a^^b^	90.08^a^^b^	42.34	81.32^a^^b^^c^	57.94^a^^b^^c^
SEM^4^		0.155	0.383	0.612	1.312	1.447	0.309	0.849	0.459
*P* value		0.660	0.827	0.719	0.023	0.007	0.810	0.005	0.002
Contrast	NC vs. PC	0.380	0.363	0.217	0.645	0.002	0.193	0.008	0.009
	NC vs. NC+POL	0.159	0.862	0.273	0.299	0.007	0.409	0.179	0.235
	NC vs. NC+CET	0.522	0.844	0.523	0.125	0.055	0.372	0.719	0.864
	NC vs. NC+POL+TLL	0.780	0.659	0.772	0.361	0.018	0.972	0.248	0.850
	NC vs. NC+POL+CRL	0.977	0.384	0.267	0.891	0.000	0.179	0.003	0.003
	NC vs. NC+CET+CRL	0.705	0.911	0.745	0.003	0.033	0.642	0.575	0.493
	NC vs. NC+LL	0.129	0.831	0.294	0.511	0.186	0.539	0.601	0.724

1 *Values are the mean of 6 replicates per treatment*.

2 *PC, Positive control diet with standard nutrient levels; NC, Negative control with 100 kcal reduce energy diet; POL, Polysorbate-20; CET, Ceteth-20; TLL, Thermomyces lanuginosus lipase; CRL, Candida rugosa lipase*.

3 a-c *Means within a same column with no common superscript differ significantly (P < 0.05)*.

4 *Standard error of mean*.

No difference (*P* > 0.05) was found in feed intake of broiler chickens among dietary treatments from hatch to 35 days of age (Table 5).

**Table 5:** Effect of emulsifier and lipase supplementation in diet on feed intake (g/d) of broiler chickens^1^^,^^2^.

**Treatment**		**Day 7**	**Day 14**	**Day 21**	**Day 28**	**Day 35**	**Day 1–21**	**Day 22–35**	**Day 1-35**
PC		28.73	53.67	98.90	124.93	185.45	60.44	155.19	98.34
NC		27.71	53.10	93.01	125.41	185.05	57.10	155.23	96.85
NC+POL		28.55	54.06	92.75	121.55	188.29	58.45	154.92	97.04
NC+CET		27.58	53.70	94.42	124.90	186.62	59.39	155.76	97.44
NC+POL+TLL		27.27	52.32	92.23	121.62	184.04	57.11	152.83	95.49
NC+POL+CRL		28.11	54.94	95.68	123.72	188.20	59.58	155.96	98.13
NC+CET+CRL		27.04	52.44	95.54	124.47	193.44	58.34	158.96	98.59
NC+LL		26.94	51.34	91.64	122.46	184.23	56.64	153.35	95.32
SEM^3^		0.233	0.474	0.660	0.764	1.384	0.360	0.852	0.434
*P* value		0.418	0.682	0.104	0.845	0.760	0.061	0.758	0.434
Contrast	NC vs. PC	0.279	0.768	0.073	0.880	0.945	0.075	0.990	0.398
	NC vs. NC+POL	0.371	0.625	0.918	0.232	0.574	0.310	0.927	0.916
	NC vs. NC+CET	0.894	0.760	0.573	0.872	0.785	0.123	0.879	0.736
	NC vs. NC+POL+TLL	0.638	0.689	0.757	0.240	0.861	0.996	0.485	0.438
	NC vs. NC+POL+CRL	0.667	0.349	0.290	0.597	0.585	0.067	0.833	0.467
	NC vs. NC+CET+CRL	0.478	0.735	0.314	0.768	0.150	0.349	0.281	0.323
	NC vs. NC+LL	0.411	0.369	0.586	0.358	0.887	0.723	0.583	0.381

1 *Values are the mean of 6 replicates per treatment*.

2 *PC, Positive control diet with standard nutrient levels; NC, Negative control with 100 kcal reduce energy diet; POL, Polysorbate-20; CET, Ceteth-20; TLL, Thermomyces lanuginosus lipase; CRL, Candida rugosa lipase*.

3 *Standard error of mean*.

The effect of emulsifier and lipase supplementation on feed efficiency of the broiler chickens from hatch to 35 days of age is present in Table 6. There was no dietary effect (*P* > 0.05) on the feed efficiency of broiler chickens from hatch to 21 days of age. Nevertheless, feed efficiency was affected (*P* < 0.05) by dietary treatment on day 28 and 35. Moreover, during the grower period from 21 to 35 days and the overall period from 0 to 35 days, improved (*P* < 0.05) feed conversion efficiency was observed with the birds fed NC+POL+CRL diet whereas birds fed NC+CER+CRL diet showed the worst (*P* < 0.05) feed conversion efficiency.

**Table 6:** Effect of emulsifier and lipase supplementation in diet on feed conversion efficiency of broiler chickens^1^^,^^2^^,^^3^.

**Treatment**		**Day 7**	**Day 14**	**Day 21**	**Day 28**	**Day 35**	**Day 1-21**	**Day 22-35**	**Day 1-35**
PC		1.74	1.25	1.42	1.63^a^	1.86^a^	1.47	1.74^a^	1.60^a^^b^
NC		1.62	1.28	1.36	1.68^a^	2.23^b^	1.42	1.96^a^^b^	1.69^a^^b^
NC+POL		1.76	1.29	1.33	1.73^a^^b^	1.94^a^^b^	1.46	1.83^a^^b^	1.64^a^^b^
NC+CET		1.65	1.30	1.44	1.85^a^^b^	2.01^a^^b^	1.45	1.93^a^^b^	1.71^a^^b^
NC+POL+TLL		1.61	1.28	1.46	1.74^a^^b^	1.93^a^^b^	1.41	1.83^a^^b^	1.66^a^^b^
NC+POL+CRL		1.64	1.27	1.38	1.63^a^	1.81^a^	1.43	1.72^a^	1.58^a^
NC+CET+CRL		1.60	1.25	1.42	2.07^b^	2.05^a^^b^	1.42	2.06^b^	1.75^b^
NC+LL		1.69	1.25	1.32	1.70^a^^b^	2.08^a^^b^	1.42	1.89^a^^b^	1.65^a^^b^
SEM^4^		0.020	0.012	0.016	0.033	0.030	0.011	0.020	0.013
*P* value		0.383	0.945	0.264	0.011	0.008	0.872	0.002	0.017
Contrast	NC vs. PC	0.148	0.588	0.317	0.671	0.001	0.326	0.012	0.068
	NC vs. NC+POL	0.078	0.750	0.731	0.691	0.009	0.363	0.131	0.311
	NC vs. NC+CET	0.695	0.633	0.181	0.153	0.045	0.611	0.756	0.673
	NC vs. NC+POL+TLL	0.934	0.924	0.101	0.600	0.006	0.850	0.131	0.528
	NC vs. NC+POL+CRL	0.757	0.975	0.751	0.671	0.000	0.831	0.005	0.025
	NC vs. NC+CET+CRL	0.853	0.656	0.317	0.002	0.099	0.976	0.197	0.198
	NC vs. NC+LL	0.411	0.588	0.615	0.876	0.150	0.935	0.387	0.461

1 *Values are the mean of 6 replicates per treatment*.

2 *PC, Positive control diet with standard nutrient levels; NC, Negative control with 100 kcal reduce energy diet; POL, Polysorbate-20; CET, Ceteth-20; TLL, Thermomyces lanuginosus lipase; CRL, Candida rugosa lipase*.

3 a, b *Means within a same column with no common superscript differ significantly (P < 0.05)*.

4 *Standard error of mean*.

### Blood Parameters

Addition of emulsifier alone or together with lipase did not affect (*P* > 0.05) blood cholesterol, creatinine, lipase or try glyceride level in broiler chickens on 21 and 35 days of age (Table 7).

**Table 7:** Effect of emulsifier and lipase supplementation in diet on blood metabolites of the broiler chickens^1^^,^^2^.

**Treatment**		**Day 21**				**Day 35**			
		**Cholesterol, mg/dL**	**Creatinine, mg/dL**	**Lipase, U/L**	**Try glyceride, mg/dL**	**Cholesterol, mg/dL**	**Creatinine, mg/dL**	**Lipase, U/L**	**Try glyceride, mg/dL**
PC		144.02	0.28	12.83	43.27	144.60	0.22	10.83	54.42
NC		143.12	0.29	14.38	38.23	138.90	0.21	12.27	43.68
NC+POL		131.80	0.30	17.08	34.33	134.93	0.24	12.50	57.13
NC+CET		134.93	0.32	14.40	41.10	134.83	0.23	10.87	44.28
NC+POL+TLL		148.42	0.29	15.13	44.68	136.45	0.21	13.00	53.23
NC+POL+CRL		144.54	0.31	16.96	38.57	142.55	0.26	12.60	54.20
NC+CET+CRL		129.75	0.28	14.48	31.37	143.05	0.23	10.80	53.65
NC+LL		126.86	0.31	13.87	34.28	139.68	0.21	11.10	50.20
SEM^3^		2.144	0.005	0.474	1.329	1.732	0.005	0.325	1.842
*P* value		0.082	0.487	0.409	0.166	0.786	0.101	0.447	0.533
Contrast	NC vs. PC	0.909	0.801	0.429	0.318	0.431	0.929	0.257	0.157
	NC vs. NC+POL	0.156	0.403	0.176	0.438	0.583	0.114	0.868	0.078
	NC vs. NC+CET	0.302	0.158	0.993	0.568	0.574	0.329	0.269	0.936
	NC vs. NC+POL+TLL	0.502	0.998	0.670	0.254	0.734	0.771	0.578	0.207
	NC vs. NC+POL+CRL	0.863	0.358	0.168	0.947	0.614	0.086	0.800	0.165
	NC vs. NC+CET+CRL	0.095	0.867	0.955	0.176	0.566	0.477	0.247	0.188
	NC vs. NC+LL	0.065	0.212	0.769	0.432	0.914	0.789	0.355	0.386

1 *Values are the mean of 6 replicates per treatment*.

2 *PC, Positive control diet with standard nutrient levels; NC, Negative control with 100 kcal reduce energy diet; POL, Polysorbate-20; CET, Ceteth-20; TLL, Thermomyces lanuginosus lipase; CRL, Candida rugosa lipase*.

3 *Standard error of mean*.

### Intestinal Organ Weights

The effect of dietary emulsifier and lipase supplementation on intestinal organ weights are presented in Table 8. No differences were found (*P* > 0.05) in the pancreas, gizzard, liver, spleen, and bursa weights of the broiler chickens fed low energy diet supplemented with emulsifier alone or together with lipase on day 21 and 35.

**Table 8:** Effect of emulsifier and lipase supplementation in diet on visceral organ weights of the broiler chickens^1^^,^^2^.

**Treatment**		**Day 21**					**Day 35**				
		**Pancreas**	**Gizzard**	**Liver**	**Spleen**	**Bursa**	**Pancreas**	**Gizzard**	**Liver**	**Spleen**	**Bursa**
PC		0.34	1.52	2.50	0.09	0.22	0.20	1.14	2.26	0.09	0.22
NC		0.34	1.71	2.55	0.08	0.24	0.22	1.10	2.16	0.11	0.20
NC+POL		0.37	1.80	2.30	0.09	0.26	0.21	1.07	2.08	0.09	0.22
NC+CET		0.33	1.77	2.41	0.10	0.24	0.20	1.08	2.27	0.11	0.22
NC+POL+TLL		0.32	1.63	2.60	0.09	0.22	0.21	1.05	2.18	0.10	0.21
NC+POL+CRL		0.33	1.63	2.35	0.07	0.25	0.22	1.11	2.17	0.09	0.24
NC+CET+CRL		0.31	1.73	2.32	0.09	0.22	0.23	1.03	2.34	0.13	0.20
NC+LL		0.31	1.85	2.46	0.08	0.22	0.20	1.10	2.10	0.09	0.21
SEM^3^		0.006	0.034	0.036	0.003	0.006	0.005	0.020	0.038	0.005	0.007
*P* value		0.251	0.254	0.316	0.648	0.737	0.654	0.917	0.720	0.290	0.969
Contrast	NC vs. PC	0.831	0.154	0.733	0.791	0.665	0.337	0.614	0.534	0.308	0.707
	NC vs. NC+POL	0.140	0.476	0.082	0.791	0.355	0.726	0.697	0.630	0.395	0.707
	NC vs. NC+CET	0.943	0.625	0.344	0.293	0.989	0.337	0.846	0.460	0.864	0.556
	NC vs. NC+POL+TLL	0.619	0.556	0.733	0.895	0.621	0.484	0.600	0.881	0.670	0.872
	NC vs. NC+POL+CRL	0.831	0.565	0.160	0.357	0.710	0.999	0.876	0.932	0.236	0.287
	NC vs. NC+CET+CRL	0.259	0.900	0.113	0.429	0.459	0.484	0.405	0.255	0.236	0.998
	NC vs. NC+LL	0.322	0.290	0.550	0.895	0.621	0.382	0.953	0.707	0.444	0.789

1 *Values are the mean of 6 replicates per treatment. Visceral organ weights are express as a proportion of live body weight*.

2 *PC, Positive control diet with standard nutrient levels; NC, Negative control with 100 kcal reduce energy diet; POL, Polysorbate-20; CET, Ceteth-20; TLL, Thermomyces lanuginosus lipase; CRL, Candida rugosa lipase*.

3 *Standard error of mean*.

### Jejunum Morphology

Decreased villi height (*P* < 0.05) commensurate with lower V:C ratio (*p* < 0.05) were observed in the broiler chickens fed NC diet compared to the birds fed PC diets on day 21 and 35 (Table 9). Moreover, higher (*P* < 0.05) crypt depth was observed in broiler chickens fed NC diet compared to the birds fed PC diet. Addition of polysorbate-20 into NC diet alone (NC+POL) or together with CRL (NC+POL+CRL) lowered (*P* < 0.05) the crypt depth and increased V:C ratio of the broiler chickens compared to the birds fed other dietary treatments on day 21. Similarly, lowered (*P* < 0.05) crypt depth and increased (*P* < 0.05) V:C ratio were observed in broiler chickens fed NC+POL and NC+POL+CRL diets compared to other dietary treatments on day 35. Nevertheless, no emulsifier or lipase effect was observed in the villi width of the broiler chickens either on day 21 or day 35.

**Table 9:** Effect of emulsifier and lipase supplementation in diet on jejunal morphology of the broiler chickens^1^^,^^2^^,^^3^.

**Treatment**		**Day 21**				**Day 35**			
		**Villi height (μm)**	**Crypt depth (μm)**	**Villi width (μm)**	**V:C ratio**	**Villi height**	**Crypt depth**	**Villi width**	**V:C ratio**
PC		1029.18^b^	97.23^b^^c^^d^	89.83	10.94^b^^c^^d^	1137.29^b^	94.59^b^^c^^d^	93.35	12.05^a^^b^^c^
NC		857.50^a^	102.37^c^^d^	97.67	8.78^a^^b^	959.32^a^	109.12^d^	93.00	9.332^a^
NC+POL		932.10^a^^b^	75.65^a^^b^	89.83	12.72^de^	1020.81^a^^b^	77.05^a^	96.12	13.66^c^
NC+CET		879.20^a^	112.88^d^	88.53	8.45^a^	932.99^a^	94.68^b^^c^^d^	94.82	10.03^a^^b^
NC+POL+TLL		976.34^a^^b^	87.17^a^^b^^c^	96.26	11.52^c^^d^	1057.50^a^^b^	85.20^a^^b^^c^	95.43	12.60^b^^c^
NC+POL+CRL		1011.63^b^	69.69^a^	92.85	14.87^e^	1057.15^a^^b^	80.56^a^^b^	102.14	13.29^c^
NC+CET+CRL		892.90^a^	104.89^c^^d^	90.21	9.45^a^^b^^c^	931.82^a^	97.98^c^^d^	116.24	9.58^a^
NC+LL		956.44^a^^b^	98.09^c^^d^	92.83	9.68^a^^b^^c^	1009.19^a^^b^	79.41^a^^b^	95.41	12.80^b^^c^
SEM^4^		14.864	2.610	1.083	0.350	16.862	2.022	2.775	0.327
*P* value		0.020	0.001	0.317	0.001	0.015	0.001	0.406	0.001
Contrast	NC vs. PC	0.003	0.463	0.081	0.005	0.003	0.007	0.974	0.004
	NC vs. NC+POL	0.156	0.001	0.068	0.001	0.304	0.001	0.780	0.001
	NC vs. NC+CET	0.676	0.120	0.085	0.638	0.678	0.015	0.879	0.483
	NC vs. NC+POL+TLL	0.027	0.027	0.737	0.001	0.104	0.001	0.828	0.001
	NC vs. NC+POL+CRL	0.005	0.001	0.254	0.001	0.090	0.001	0.393	0.001
	NC vs. NC+CET+CRL	0.517	0.718	0.096	0.357	0.628	0.035	0.084	0.778
	NC vs. NC+LL	0.062	0.521	0.253	0.200	0.381	0.001	0.821	0.001

1 *Values are the mean of 6 replicates per treatment*.

2 *PC, Positive control diet with standard nutrient levels; NC, Negative control with 100 kcal reduce energy diet; POL, Polysorbate-20; CET, Ceteth-20; TLL, Thermomyces lanuginosus lipase; CRL, Candida rugosa lipase*.

3 a-d *Means within a same column with no common superscript differ significantly (P < 0.05)*.

4 *Standard error of mean*.

### Nutrient Digestibility

The ileal digestibility of dry matter, crude protein, crude fat, and energy in response to dietary emulsifier and lipase supplementation are shown in Table 10. At the end of the starter period (day 21), the nutrient digestibility of the broilers was similar (*P* > 0.05) in all experimental treatments. However, higher (*P* < 0.05) fat digestibility was observed in the birds fed NC+POL and NC+POL+CRL diets compared to birds fed NC diet on day 35. In a meanwhile, the lower (*P* < 0.05) fat digestibility was shown in broiler chickens fed NC+CET+CRL diet compared to those fed the other treatment groups. Moreover, higher (*P* < 0.05) energy digestibility was shown the birds fed with NC+ POL+CRL diet compared to the broiler chickens fed NC diet on day 35. However, the dry matter digestibility and crude protein digestibility were not affected (*P* > 0.05) by the dietary treatments.

**Table 10:** Effect of emulsifier and lipase supplementation in diet on nutrient digestibility of the broiler chickens^1^^,^^2^^,^^3^.

**Treatment**		**Day 21**				**Day 35**			
		**DM**	**CP**	**CF**	**Energy**	**DM**	**CP**	**CF**	**Energy**
PC		56.03	75.38	94.82	61.94	75.07	83.86	93.87^b^^c^	77.82^a^^b^
NC		51.49	74.45	93.32	58.88	73.08	83.08	92.80^a^^b^	75.97^a^
NC+POL		58.61	76.60	94.35	64.67	75.93	83.81	94.17^c^	78.50^a^^b^
NC+CET		55.11	75.44	93.09	60.23	72.76	82.75	92.77^a^^b^	75.94^a^
NC+POL+TLL		56.63	75.32	95.63	61.87	74.53	85.20	93.23^a^^b^^c^	77.84^a^^b^
NC+POL+CRL		59.77	77.32	95.35	63.18	73.75	83.83	94.42^c^	80.27^b^
NC+CET+CRL		52.49	74.25	93.37	57.88	76.39	85.15	92.44^a^	76.92^a^^b^
NC+LL		59.24	76.63	93.78	64.69	74.83	85.87	93.70^a^^b^^c^	77.76^a^^b^
SEM^4^		0.817	0.404	0.284	0.711	0.342	0.397	0.138	0.333
*P* value		0.079	0.522	0.164	0.123	0.070	0.481	0.001	0.016
Contrast	NC vs. PC	0.144	0.572	0.176	0.263	0.124	0.626	0.014	0.123
	NC vs. NC+POL	0.025	0.195	0.666	0.038	0.030	0.651	0.002	0.038
	NC vs. NC+CET	0.243	0.544	0.120	0.619	0.804	0.839	0.943	0.982
	NC vs. NC+POL+TLL	0.100	0.594	0.463	0.273	0.259	0.191	0.310	0.120
	NC vs. NC+POL+CRL	0.010	0.085	0.629	0.119	0.597	0.641	0.001	0.001
	NC vs. NC+CET+CRL	0.744	0.903	0.190	0.714	0.013	0.202	0.403	0.424
	NC vs. NC+LL	0.015	0.187	0.346	0.037	0.176	0.088	0.037	0.136

1 *Values are the mean of 6 replicates per treatment*.

2 *DM, Dry matter; CP, Crude protein; CF, Crude fat; PC, Positive control diet with standard nutrient levels; NC, Negative control with 100 kcal reduce energy diet; POL, Polysorbate-20; CET, Ceteth-20; TLL, Thermomyces lanuginosus lipase; CRL, Candida rugosa lipase*.

3 a–c *Means within a same column with no common superscript differ significantly (P < 0.05)*.

4 *Standard error of mean*.

### Carcass Measurements

There were no effects (*P* > 0.05) between emulsifier and lipase treatments with respect to breast and leg muscle weights of broiler chickens at day 21 and day 35 (Table 11). However, a higher proportion of abdominal fat was observed in the broiler chickens fed PC diets compared to its counterpart fed NC diet on day 21. Nevertheless, this observed dietary effect on abdominal fat of broiler chickens did not remain on day 35 (*P* > 0.05).

**Table 11:** Effect of emulsifier and lipase supplementation in diet on carcass measurements of broiler chickens^1^^,^^2^^,^^3^.

**Treatment**		**Day 21**			**Day 35**		
		**Breast %**	**Leg %**	**Abdominal fat %**	**Breast %**	**Leg %**	**Abdominal fat %**
PC		23.58	8.98	0.88^b^	25.07	9.28	1.13
NC		23.03	8.60	0.47^a^	24.88	9.40	0.94
NC+POL		22.62	8.82	0.63^a^^b^	25.26	9.09	0.96
NC+CET		22.92	9.15	0.67^a^^b^	26.11	9.22	1.02
NC+POL+TLL		23.01	9.01	0.69^a^^b^	26.13	9.22	1.11
NC+POL+CRL		22.78	9.11	0.51^a^^b^	25.43	9.15	1.13
NC+CET+CRL		23.03	8.89	0.52^a^^b^	26.27	9.10	0.99
NC+LL		22.44	8.92	0.70^a^^b^	25.47	9.49	0.86
SEM^4^		0.197	0.062	0.034	0.221	0.061	0.047
*P* value		0.933	0.428	0.048	0.090	0.706	0.816
Contrast	NC vs. PC	0.509	0.128	0.002	0.835	0.637	0.344
	NC vs. NC+POL	0.622	0.379	0.221	0.677	0.213	0.945
	NC vs. NC+CET	0.895	0.080	0.118	0.181	0.466	0.679
	NC vs. NC+POL+TLL	0.981	0.101	0.091	0.175	0.462	0.394
	NC vs. NC+POL+CRL	0.763	0.062	0.783	0.547	0.312	0.328
	NC vs. NC+CET+CRL	0.998	0.239	0.713	0.134	0.227	0.802
	NC vs. NC+LL	0.484	0.200	0.076	0.521	0.734	0.685

1 *Values are the mean of 6 replicates per treatment. Carcass measurements are express as a proportion of live body weight*.

2 *PC, Positive control diet with standard nutrient levels; NC, Negative control with 100 kcal reduce energy diet; POL, Polysorbate-20; CET, Ceteth-20; TLL, Thermomyces lanuginosus lipase; CRL, Candida rugosa lipase*.

3 a, b *Means within a same column with no common superscript differ significantly (P < 0.05)*.

4 *Standard error of mean*.

### Meat Quality

Water holding capacity, pH, lightness, and redness of the broiler meat did not affect (*P* > 0.05) by the emulsifier or lipase addition into low energy diets on day 35 of the study (Table 12). However, broilers fed NC+POL+TLL diet were shown comparatively higher (*P* < 0.05) yellowness (b^*^) compared to meat from broiler chickens fed PC diet on day 35. Moreover, higher (*P* < 0.05) cooking loss was observed in the meat from broiler chickens fed NC+CET+CRL compared to the birds fed NC diets.

**Table 12:** Effect of emulsifier and lipase supplementation in diet on meat quality of broiler chickens^1^^,^^2^^,^^3^.

**Treatment**		Lightness (L*)	Redness (a*)	Yellowness (b*)	**pH**	**Cooking loss**	**WHC**
PC		54.65	5.53	14.06^a^	5.97	22.05^a^	74.52
NC		56.60	5.69	16.09^a^^b^	5.97	22.55^a^	80.57
NC+POL		55.40	4.99	14.44^a^^b^	5.97	24.09^a^^b^	78.19
NC+CET		55.98	6.10	16.17^a^^b^	5.99	25.09^a^^b^	78.24
NC+POL+TLL		56.05	5.96	16.66^b^	5.97	23.89^a^^b^	79.01
NC+POL+CRL		55.21	5.12	15.41^a^^b^	6.02	24.17^a^^b^	79.72
NC+CET+CRL		57.79	5.12	15.99^a^^b^	5.96	29.22^b^	78.94
NC+LL		58.67	4.83	14.43^a^^b^	5.92	26.85^a^^b^	76.81
SEM^4^		0.386	0.158	0.221	0.013	0.530	0.560
*P* value		0.138	0.364	0.008	0.765	0.008	0.193
Contrast	NC vs. PC	0.192	0.806	0.011	0.998	0.787	0.068
	NC vs. NC+POL	0.418	0.272	0.037	0.989	0.408	0.277
	NC vs. NC+CET	0.672	0.510	0.914	0.716	0.175	0.286
	NC vs. NC+POL+TLL	0.710	0.665	0.462	0.976	0.472	0.472
	NC vs. NC+POL+CRL	0.348	0.369	0.378	0.366	0.384	0.696
	NC vs. NC+CET+CRL	0.423	0.375	0.900	0.809	0.001	0.453
	NC vs. NC+LL	0.167	0.180	0.035	0.320	0.024	0.089

1 *Values are the mean of 6 replicates per treatment*.

2 *PC, Positive control diet with standard nutrient levels; NC, Negative control with 100 kcal reduce energy diet; POL, Polysorbate-20; CET, Ceteth-20; TLL, Thermomyces lanuginosus lipase; CRL, Candida rugosa lipase*.

3 a, b *Means within a same column with no common superscript differ significantly (P < 0.05)*.

4 *Standard error of mean*.

## Discussion

Owing to the increase in the price of cereal grains in recent years, the addition of fat to practical diet formulations has become popular. However, it has been reported that endogenous emulsifiers alone do not support proper fat digestion in poultry (6). Exogenous emulsifiers are capable of improving fat digestibility and subsequently sustaining or enhancing the growth performance of broiler chickens fed a low-density energy diet (5). With this conviction, studies on different exogenous emulsifiers were conducted to determine their effect on the improvement in the growth performance of broiler chickens (9, 21, 22). Our current study tested the hypothesis that emulsifiers alone, or together with lipase would improve or sustain growth performance by enhancing gut integrity and nutrient digestibility of the birds fed a low-energy diet.

According to Meng et al. (2), beef tallow showed poor digestibility in chickens, which rely on the adequate presence of bile salts for efficient emulsification and micelle formation. Ward and Marquardt (23) attributed such diminished digestibility of tallow to the degree of saturation of its fatty acids. Hence, in this experiment, beef tallow was incorporated into the diet at a rate of 4.5% with the aim of providing a suitable substrate for the catalytic action of exogenous emulsifier supplements to broiler diets. In this experiment, lipases were tested together with emulsifiers because of their known effect on dietary fat utilization efficacy in broiler chickens (4). Lipases hydrolyze triglycerides into fatty acids and promote the formation of micelles for fat digestion and absorption through the intestinal mucosa (24).

In this study, broiler chickens fed a low-energy diet supplemented with emulsifiers and lipases did not show improved growth performance or feed efficiency than those fed negative control (NC) and positive control (PC) diets in the starter phase (0–3 weeks). This observation confirmed that the secretion of endogenous lipase is less pronounced when calculated per gram of feed intake, although the net secretion increases with bird age (25). Moreover, it was possible that the intestinal physiology or the fat absorption capacity in the early stages of growth was the same in all birds receiving all treatments. Therefore, fat absorption was similar to all diets, even though some dietary treatments provided more fatty acids for the digestive process. This may be the reason for the non-significant difference in fat digestibility in the starter phase observed in our study. Therefore, birds fed PC and NC diets also have the same fatty acid absorption owing to the same amount of tallow in all treatment diets. Interestingly, the energy difference between NC and PC diets is attributed to the difference in starch levels in each diet. Based on this, we can argue that birds receiving PC diets should show higher overall performance than those receiving NC diets. However, it has been found that glucose is the primary source of energy for enterocytes and is less likely to bypass them. Therefore, glucose cannot contribute significantly to the weight gain of broilers, especially at an early age (26).

In the present study, improved growth performance was observed in birds receiving a low-energy diet supplemented only with polysorbate-20 (NC+POL) or POL with CRL (NC+POL+CRL) during week 4 and 5 and during the overall period (0–35 days). In contrast, birds fed a low-energy diet supplemented only with Ceteth-20 or Ceteth-20 with CRL showed lower growth performance. This suggests that different emulsifiers perform differently in the same diet for broilers. The reason for the lower growth performance observed with Ceteth-20 maybe its toxicity that lowered nutrition absorption *via* the intestinal wall. The effects of these Ceteth applications have been previously reviewed (15), and reported the irritant effects that caused skin damage in humans and in some rabbit models. However, the effects of supplementing animal diets with Ceteth has not been reported, and the underlying mechanism is not clear.

Siyal et al. (27) reported that the synthetic exogenous emulsifiers used in broiler diets did not affect feed intake. This is in agreement with the present study that confirmed that feed intake was not affected by the addition of emulsifiers and lipases to low-energy diets. Moreover, the feed intake observed in our study is supported by that in previous studies (3, 21), which also found no difference in feed intake among dietary treatments with different emulsifiers.

The improvement of feed efficiency with the addition of exogenous emulsifiers and lipases, without the alteration of feed intake during the grower period of this study, corresponded to the daily gain improvement and may have been because of improved nutrient digestibility during this period. Similarly, in previous broiler studies, an improvement in feed efficiency without the alteration of feed intake was observed with emulsifier supplementation to the diet (9, 10, 28). However, in contradiction to our results, two studies (29, 30) reported that the addition of exogenous emulsifiers or lipase did not affect the feed efficiency of broiler chickens. Diet, fat source, and emulsifier interaction-driven effects may be the reason for the observed differences in outcome between the results of the present study and those of previous studies.

Elevated serum lipids, such as cholesterol and triglycerides, due to the inclusion of animal fat in broiler diets can be efficiently reduced by the inclusion of emulsifiers (9). Supporting this finding, Zhao and Kim (28) reported that lysophospholipid supplementation reduced total cholesterol, LDL, and triglycerides in broiler chickens fed tallow incorporated into corn soy-bean diets. Similarly, another study observed reduced total cholesterol, LDL, and triglyceride profiles in broiler chickens fed emulsifier-supplemented broiler diets (13, 21). In the present study, the responses of all blood metabolites at 21 and 35 days of age did not differ between the dietary treatments. Similarly, Guerreiro Neto et al. (3) and Upadhaya et al. (9) reported that emulsifier supplementation did not affect the blood metabolites of broiler chickens at days 42 and 35, respectively. The observed differences in results may be ascribed to the consequences of the interactions between metabolism and absorption mechanisms.

According to previous studies, emulsifier supplementation did not affect the visceral organ weight of broiler chickens (3, 21, 31, 32). This is in agreement with the finding of the present study that emulsifier and/or lipase supplementation did not influence the proportion of visceral organ weights.

Intestinal mucosal morphology is considered a biomarker of gut health and is an indicator of the nutrition absorption capacity of broiler chickens (33, 34). Greater villi height, associated with increased surface area, allows higher nutrient absorption (13). Our results show that on day 21, only polysorbate-20 supplementation to the NC diet (NC+POL), or together with CRL (NC+POL+CRL) significantly lowered the crypt depth and increased the V:C ratio; commensurate with increased villi height of the broiler chickens compared to the birds fed other dietary treatments. Similarly, on day 35, significantly lowered crypt depth and increased V:C ratio was observed in broiler chickens fed NC+POL and NC+POL+CRL diets compared to other dietary treatments. Consistent with our results, Hu et al. (13) reported an increased jejunum villi height along with an increased V:C ratio on day 28, in broiler chickens fed a low-energy, tallow-incorporated diet, supplemented with heat-resistant lipase. In contrast, Boontiam et al. (33) did not observe a significant difference in jejunum villi height and crypt depth on day 35, in broiler chickens fed a lysophospholipid-supplemented diet containing soybean oil. The observed differences in results may be ascribed to the differences in interactions between dietary fat type and the emulsifiers or lipases utilized in those experiments.

In general, the addition of emulsifiers or lipase to a low-energy diet enhanced the nutrient digestibility of the broilers. However, a number of factors may contribute to the utilization of supplemental fat, including the composition of the supplemental fat, the physical form of the fat, and the quantity of the emulsifiers (35). The level of dietary fat may also influence the effectiveness of exogenous emulsifiers in aiding digestion (36). In this study, the nutrient digestibility results on day 35 supported the observed growth performance, and reflect that the addition of Polysorbate-20 and CRL to low-energy diets (NC+POL+CRL) enhances fat and energy digestibility of the growing broilers. These observations are supported by previous studies (3, 29, 32), which showed improved energy and fat digestibility of broiler chickens. More interestingly, the addition of Ceteth-20 to a low-energy diet (NC+CET) lowered the fat and energy digestibility of the growing broilers. This may be the reason for the observed lower growth performance of the NC+CET-supplemented diet fed to broilers at the grower stage. Nevertheless, the exact reason for this lowered energy digestibility is not clear, nor has it been previously reported.

In accord with consumer demand for healthy and portioned meat, higher meat yield and lower abdominal fat are considered important parameters in the broiler industry (37). In the present study, on days 21 and 35, birds fed a diet supplemented either only with an emulsifier or with emulsifier and lipase, showed no difference in breast or leg meat yield. Moreover, on day 35, emulsifier alone or together with lipase supplementation did not affect the abdominal fat yield. Nevertheless, the addition of emulsifiers and lipases to a low-energy diet tended to increase abdominal fat yield in broiler chickens on day 21. Consistent with our results, earlier studies (3, 21) reported that the addition of exogenous emulsifiers to broiler diets did not affect the carcass and abdominal fat yield of broiler chickens. In contrast, Zhao and Kim (28) reported that the addition of lysophospholipids to tallow-incorporated diets reduced the abdominal fat pad yield of broiler chickens on day 28, regardless of the dietary energy level. On the other hand, Wang et al. (22) observed that the addition of sodium stearoyl-2-lactylate to a reduced energy diet increased the abdominal fat yield of broiler chickens on day 35. The paradox that the abdominal fat yield results are not univocal in the different studies may be ascribed to the consequences of the interactions between the different emulsifiers, inclusion levels, fat sources, and sampling ages (28).

The expectation of improved meat quality is an ever-rising trend of poultry consumption that underscores the importance of controlling meat quality in the poultry industry (38). Water holding capacity, pH, color, and tenderness are the primary determinants of meat quality and are crucial for the culinary value and technological properties of chicken meat (39, 40). Many nutritional studies have reported the importance of the dietary effects on the meat quality of broilers. In this study, the water holding capacity and pH of broiler breast meat were not affected by the dietary emulsifier and lipase additions to low-energy diets. Moreover, the dietary treatments in this study did not affect the lightness of the color and redness of the meat. Consistent with our results, earlier studies (28, 41) have reported that the addition of exogenous emulsifiers to broiler diets did not significantly affect the water holding capacity, pH, lightness, and redness of broiler meat. Yellowness of the meat reflects the myoglobin concentration and its redox states, which is positively related to the overall sensory properties of the meat color (42). Bontempo et al. (41) reported that supplementation with a synthetic emulsifier product consisting of a vegetal bi-distilled oleic acid and glycerol polyethylene glycolricinoleate increased the yellowness of breast meat of broiler chickens on day 44. In support of this finding, on day 35, we observed increased breast muscle yellowness in the broiler chickens fed the NC+POL+TLL diet. Similarly, it was noted (42) that the inclusion of emulsifiers increased the yellowness of broiler meat, while the addition of emulsifier together with essential oils decreased the yellowness. This observation can be ascribed to the role of the emulsifier in increasing the accumulation of lipid-soluble pigments, such as xanthophyll, in breast muscle (41). In the present study, broiler chickens fed the NC+CET+CRL diet had a significantly higher cooking loss in breast meat compared to birds fed the NC diet. In contrast, other studies (6, 42) did not observe a dietary emulsifier effect on cooking loss of broiler meat. The exact underlying mechanism for increased cooking loss is not clear and difficult to explain.

## Conclusions

In conclusion, polysorbate-20 together with *Candida rugosa* lipases could be a suitable combination for the commercial broilers fed with low-energy diet as these combinations improved gut health, increased fat, and energy digestibility commensurate with improved growth performance. However, it is necessary to study the effects of Ceteth-20 to gain a better understanding of the mechanisms of reduced-fat and energy digestibility, and growth depression in broilers.

## Data Availability Statement

The raw data supporting the conclusions of this article will be made available by the authors, without undue reservation.

## Ethics Statement

The animal study was reviewed and approved by Animal ethics committee of the Chungnam National University (Protocol No. CNU-00863).

## Author Contributions

All authors listed have made a substantial, direct and intellectual contribution to the work, and approved it for publication.

## Conflict of Interest

SP was employed by the company SNH Biotech Co., Ltd. The remaining authors declare that the research was conducted in the absence of any commercial or financial relationships that could be construed as a potential conflict of interest.
